# Altered excitability of small cutaneous nerve fibers during cooling assessed with the perception threshold tracking technique

**DOI:** 10.1186/s12868-019-0527-3

**Published:** 2019-09-03

**Authors:** Rosa Hugosdottir, Carsten Dahl Mørch, Cecilia Klitgaard Jørgensen, Camilla Winther Nielsen, Mathias Vassard Olsen, Mads Jozwiak Pedersen, Jenny Tigerholm

**Affiliations:** Center of Neuroplasticity and Pain, SMI®, Department of Health Science and Technology, Aalborg University, Fredrik Bajers Vej 7D3, 9220 Aalborg, Denmark

**Keywords:** Electrical stimulation, Perception threshold tracking, Voltage-gated ion channels, Na/K-ATPase, Multicompartment model, Temperature

## Abstract

**Background:**

There is a need for new approaches to increase the knowledge of the membrane excitability of small nerve fibers both in healthy subjects, as well as during pathological conditions. Our research group has previously developed the perception threshold tracking technique to indirectly assess the membrane properties of peripheral small nerve fibers. In the current study, a new approach for studying membrane excitability by cooling small fibers, simultaneously with applying a slowly increasing electrical stimulation current, is evaluated. The first objective was to examine whether altered excitability during cooling could be detected by the perception threshold tracking technique. The second objective was to computationally model the underlying ionic current that could be responsible for cold induced alteration of small fiber excitability. The third objective was to evaluate whether computational modelling of cooling and electrical simulation can be used to generate hypotheses of ionic current changes in small fiber neuropathy.

**Results:**

The excitability of the small fibers was assessed by the perception threshold tracking technique for the two temperature conditions, 20 °C and 32 °C. A detailed multi-compartment model was developed, including the ionic currents: Na_TTXs_, Na_TTXr_, Na_P_, K_Dr_, K_M_, K_Leak_, K_A_, and Na/K-ATPase. The perception thresholds for the two long duration pulses (50 and 100 ms) were reduced when the skin temperature was lowered from 32 to 20 °C (p < 0.001). However, no significant effects were observed for the shorter durations (1 ms, p = 0.116; 5 ms p = 0.079, rmANOVA, Sidak). The computational model predicted that the reduction in the perception thresholds related to long duration pulses may originate from a reduction of the K_Leak_ channel and the Na/K-ATPase. For short durations, the effect cancels out due to a reduction of the transient TTX resistant sodium current (Na_v_1.8). Additionally, the result from the computational model indicated that cooling simultaneously with electrical stimulation, may increase the knowledge regarding pathological alterations of ionic currents.

**Conclusion:**

Cooling may alter the ionic current during electrical stimulation and thereby provide additional information regarding membrane excitability of small fibers in healthy subjects and potentially also during pathological conditions.

## Background

In humans, the skin and deeper tissue is innervated by peripheral sensory neurons called nociceptors, which are responsible for detecting noxious stimuli and conveying nociceptive information to the central nervous system. During pathological conditions, nociceptors’ excitability can be increased, which can amplify sensations to noxious stimuli, and thus cause a chronic pain condition [1, 2]. To study the excitability in nociceptors directly is technically challenging due to the small diameter of the fibers. Therefore, small area cathodes have been developed to study small fibers in isolation, with limited activation of the larger fibers [3–10] by generating high current density in epidermis where the small fibers terminate, unlike the larger non-nociceptive fibers which terminate in dermis [11–16]. Non-invasive pin electrodes, with small surface area cathodes, predominately activate the small-diameter Aδ fibers, however co-activation of C-fibers cannot be ruled out [5, 9]. In large fibers, excitability of the nerve fibers can be assessed by measuring the compound action potential. For small fibers, the compound action potential is technically challenging to record. To overcome this problem, our research group has previously developed the perception threshold tracking (PTT) technique to indirectly assess the excitability of small fibers [10, 17].

Cooling the skin has mainly been used to study cool-sensitive transduction channels such as the TRPM8. However, reduced temperature also affects the dynamics of ionic currents that are not gated by cold temperature per se. In the current study, the influence of reduced temperature on nerve fiber excitability is investigated, with as little influence as possible from cool-sensitive transduction channels within sensory terminals. Instead, our focus is on the effect of cooling on other ionic currents in the peripheral part of small fibers by means of cutaneous electrical stimulation. Cutaneous electrical stimulation has an advantage over laser stimulation as it is easier to control and bypasses the sensory transduction within the sensory terminal and activates voltage-gated ion channels by changing the potential across the cell membrane. There is a wide range of subtypes of voltage-gated ion channels expressed in small fibers and many of them have been identified as candidates for altering the membrane excitability in small fiber neuropathy [18–22]. Each subtype of voltage-gated ion channels has its unique dynamics and represents a different aspect of excitability. In the Zimmerman et al. study [23] they showed that certain subtypes of transient sodium channels, TTX resistant (Na_TTXr_, Na_v_1.8) and TTX sensitive (Na_TTXs_, Na_v_1.7), were affected differently by cooling. Most voltage-gated ion channels are inactivated by cold temperatures. Although, not the Na_TTXr_ sodium channel which is resistant to cooling-induced inactivation, giving these channels a prominent role in the transduction of nociceptive signals during cooling, since they are selectively expressed in nociceptors [23, 24].

Furthermore, altered axonal excitability during cooling has also been detected in threshold tracking experiments in large fibers, as well as in small dorsal root ganglia soma [25–28]. Different ionic currents have been proposed as candidates for causing such alterations, for instance the potassium leak channel  (K_Leak_), the Na/K-ATPase and the A-type potassium channel (K_A_) [25–28]. Both the two potassium channels, K_A_ and K_Leak_ are highly expressed in dorsal root ganglia soma [29, 30]. The K_Leak_ channels, TREKs and TRESK may together generate over 95% of the background potassium current in dorsal root ganglia soma [31].

Despite the progress in recent years, there is still a need for new approaches to increase the knowledge of membrane excitability of small fibers both in healthy subjects, as well as during pathological conditions. Therefore, new approaches such as cutaneous cooling could be used to study nerve fiber excitability simultaneously with applying an electrical stimulation current. The intention of cooling the skin was not to activate cool-sensitive transduction channels but to alter the dynamics of other ionic currents, such as voltage-gated ion channels.

The first objective was to examine whether altered excitability during cooling could be detected by the PTT technique. The second objective was to computationally model the underlying ionic current that could be responsible for cold induced alteration of small fiber excitability. The third objective was to evaluate whether computational modelling of cooling and electrical simulation can be used to generate hypothesis of ionic current changes in small fibers neuropathy.

## Methods

The current study is a combination of an experimental study and computational modeling. In the experimental study, the change of perception threshold related to cooling the skin simultaneously with applying cutaneous electrical stimulation has been estimated. In the computational study, the results from the experimental study were reproduced and the influence of different ionic subtypes investigated.

### Experimental study

#### Subjects

26 healthy human subjects (14 female and 12 male) were recruited to participate in an experimental study. Exclusion criteria for the subjects were neurological diseases, injury to, or disease on, the volar forearms, chronic pain or influence of analgesic substances. One subject was excluded, due to a musculoskeletal disorder, resulting in a total of 25 participants (13 females and 12 males, with an average age of 22.7 ± 1.3 years). The experimental procedure was approved by the local ethics committee (Den Videnskabsetiske Komité, Region Nordjylland, approval number: N-20160076), and the study was conducted according to the Declaration of Helsinki. The subjects gave their written informed consent to the experimental procedure prior to participation in the study.

#### Experimental setup

**Fig. 1:**
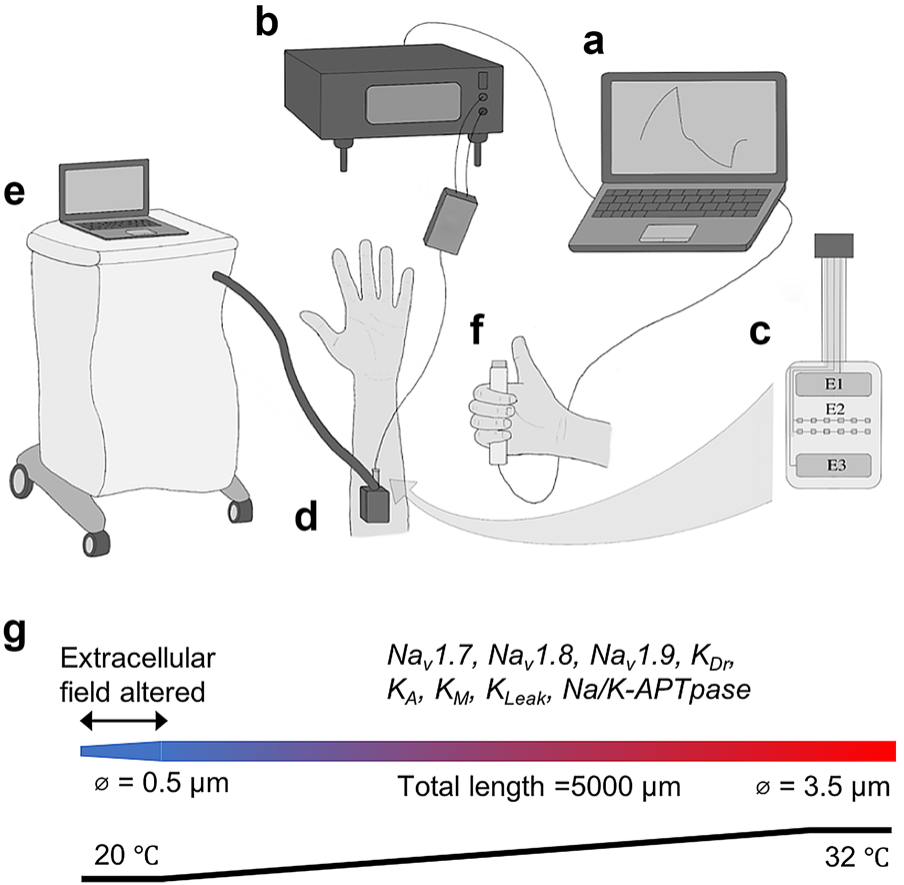
The experimental setup and the computational model design. A computer (**a**) was used to control the pulses delivered by the Digitimer DS5 stimulator (**b**) which electrically stimulated through the custom-made planer array electrode (**c**), that was placed on the volar forearm of the subject. A thermode (**d**) from the Pathway system, used for controlling the temperature, was placed on top of the electrode (**e**). In the other hand the subject held a push button (**f**) which was used to indicate the perception threshold. Computational model design (**g**)

The subjects were placed in a comfortable position in a chair, with the arm resting in front of them on a soft surface. The subjects were electrically stimulated through a custom-made planer array electrode (size: 40 × 34 × 0.2 mm), see Fig. 1c. This electrode array consisted of three electrode areas (E1, E2 and E3). E2 consisted of 12 small quadratic electrode sites (size: 0.6 × 0.6 mm), which functioned as small area cathodes for the electrode. The electrode used the small area cathodes (E2) to activate the smaller nerve fibers. The conductive areas E1 and E3 (size: 5 × 24 mm) served as anodes.

The experimental setup can be seen in Fig. 1. The planer array electrode was placed on the volar forearm of the subject, approximately 5 cm distal to the cubital fossa. It was fastened with micropore tape to secure the electrode from moving during the experiment. A 3 × 3 cm thermode (Pathway, Medoc Ltd., Ramat Yishai, IL) was placed on top of the electrode and fastened to the arm of the subject with a velcro band. In the opposite hand, the subjects held a custom-made response button (Sensory Motor-Interaction, Aalborg University), which was used to indicate when they perceived the electrical stimulation. The electrical stimuli were administered using a DS5 electrical stimulator (Digitimer Ltd., Welwyn Garden City, UK), which was controlled by a computer with an implementation of LabVIEW (Aalborg University, Aalborg, Denmark). The computer and a data acquisition card (National Instruments, Austin, Texas) collected the responses from the subjects.

#### Pulse form and duration

**Fig. 2:**
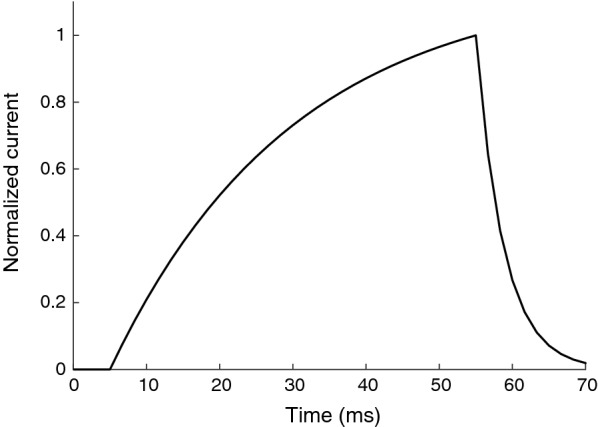
The shape of a 50 ms bounded exponential pulse. For the three other pulses, the x-axis was scaled to 1, 5 and 100 ms respectively

The pulse form used was a bounded exponential current increase, i.e. a current with an increasing form of exponential decay. This pulse form was recently shown to activate small nociceptive fibers more selectively than rectangular pulses [17]. The perception threshold was identified for four durations of this pulse form (1, 5, 50 and 100 ms). The pulse form with a duration of 50 ms is illustrated in Fig. 2 and the mathematical expression for the pulse shape is shown in Eq. 1. A charge-balancing phase of half the stimulation amplitude was added to avoid risk of damages to the skin [32].

1 $${\text{I}}\left( {\text{t}} \right) = \left\{ {\begin{array}{ll} {\frac{{{\text{I}}_{\text{s}} }}{{1 - {\text{e}}^{{\frac{{ - {\text{T}}_{\text{S}} }}{\uptau}}} }}\left( {1 - {\text{e}}^{{\frac{{ - {\text{t}}}}{\uptau}}} } \right),} & \quad {0 \le {\text{t}} < {\text{T}}_{\text{S}} } \\ {{\text{I}}_{\text{s}} \cdot {\text{e}}^{{\frac{{ - {\text{t}}}}{{\uptau_{\text{tr}} }}}} ,} & \quad {{\text{T}}_{\text{S}} \le {\text{t}} \le {\text{T}}_{\text{tr}} } \\ \end{array} } \right.$$

Equation 1, the equation for the stimulation current, I(t). I_S_ is the stimulation current intensity, T_S_ is the stimulation duration, τ = T_S_/2 is the time constant, trailing phase: T_tr_ = T_S_ * 1.4 and τ_tr_ = τ/6.6.

#### Perception threshold tracking technique

To estimate the perception threshold, the method of limits was used. Initially, an initiation point in the form of a sub-threshold current intensity was found by trial and error. From the initiation point, the stimulation intensity increased in steps of 5% at a frequency of 0.5 Hz. The stimulation was terminated when the subjects pressed the response button, indicating perception of the stimuli. From this point, the stimulation intensity was increased by another 10% and subsequently decreased in steps of 5% until the subject pressed the response button indicating that the stimuli were not perceived anymore. From this point, the stimulation intensity was further decreased by 10% and then increased again in steps of 5%, whereafter the subject pressed the response button once the stimuli were perceived again. This sequence was repeated three times. The estimated perception threshold of each duration for each subject was the mean of the three current intensities at which the subjects had indicated their perception and the three current intensities at which the subjective perception had dissipated.

#### Experimental protocol

The perception thresholds of the subjects were estimated for all pulse durations in two sessions, each composed of a different temperature setting. In one session, the thermode was set to a non-painful cold temperature of 20 °C and in the other session, the thermode was set to a neutral temperature of 32 °C to emulate typical arm skin temperature. The temperature was held constant on top of the electrode for 15 min before each session and during the stimulation period to achieve a stable temperature of the skin. The order of the two temperature sessions, the arm onto which the temperature condition was applied, as well as the order of the pulse durations within each session were all randomized.

#### Data analysis

To obtain normality, the perception thresholds were log-transformed. In the result figure, the perception threshold data was back-transformed and is shown as between subject mean and standard error for each duration. A two-way repeated measure analysis of variance (rmANOVA) was performed with the perception threshold as the dependent variable and duration (1-, 5-, 50-, and 100 ms) and temperature (20 °C cold and 32 °C neutral) as independent within-subject variables (SPSS 25, IBM). The temperature of 32° was used as neutral temperature since it is the typical arm skin temperature of the lower forearm. A post hoc test with Sidak correction were used for multiple comparisons. Before performing the rmANOVA, the normal distribution of the data was examined by examining the skewness and kurtosis. The distribution of the data skewed right, which promoted a log-transformation of the data in order to meet the criteria for a rmANOVA.

### Computational model

A computational axon model was developed in the simulator environment NEURON ([33], version 7.6). In a previous study, a detailed multi-compartment model of a C-fiber with a large range of voltage-gated ion channels has been developed [34, 35]. In the current study, the C-fiber model was further developed to include a temperature dependent K_Leak_ channel.

#### Morphology and ionic currents

The model consists of a 5000 μm long unmyelinated fiber. Even though non-invasive pin electrodes preferentially activate thinly myelinated Aδ fibers, an unmyelinated axon model was developed because the Aδ fibers lose their myelin and become indistinguishable from C-fibers when they enter the epidermis [13]. The axon model is divided into two parts. The first part (500 μm) starts distally with a diameter of 0.5 μm which is linearly increased to 3.5 μm in the proximal end. The second part is a cylinder with a diameter of 3.5 μm connecting to the first part. The resting membrane potential was reduced to − 60 mV since the Aδ fibers’ resting potential is more hyperpolarized than that of the C-fibers [36]. The parameters for the morphology are listed in Table 1.

**Table 1:** Parameters of the morphology for the axon model

	Morphology parameter	References
Diameter	0.5–3.5 μm	
Capacitance	1 μF/cm^2^	[37]
Resting membrane potential	− 60 mV	[36]
Intra cellular resistance	130 Ωcm	
Total model length	5000 μm	

The transient TTX sensitive current was denoted as Na_TTXs_ and the transient TTX resistant sodium current as Na_TTXr_. The persistent sodium current is denoted Na_P_ even though it is resistant to TTX. The six voltage-gated ion channels, Na_TTXr_ (Nav 1.8), Na_TTXs_ (mainly Nav 1.7), Na_P_ (Nav 1.9), a delayed rectifier potassium channel (K_Dr_), K_A_ and a slow potassium channel (K_M_), were implemented, all of which were adopted from the C-fiber model [34]. In order to generate a delayed rectification of the action potential, the K_Dr_ channel’s voltage dependency was shifted 15 mV towards hyperpolarization. Since the Na/K-ATPase is also highly temperature dependent, it was also implemented in the model. A K_Leak_ channel has further been implemented with a temperature dependent maximal conductance. The conductance for the K_Leak_ channel was linearly reduced from its original value to 10% when the temperature was decreased from 32 to 20 °C [29]. The maximal conductance and model reference for the voltage-gated ion channels are listed in Table 2.

**Table 2:** The models of voltage-gated ion channel

	Maximal conductance (S/cm^2^)	Q10	Model references
Na_TTXr_ (Na_v_ 1.8)	0.0623	2.5	[34]
Na_p_ (Na_v_ 1.9)	3.55 * 10^−5^	2.5	[34]
Na_TTXs_ (mainly Nav 1.7)	0.0183	2.5	[34]
K_Dr_	3.40 * 10^−4^	3.3	[34]
K_M_	1.05 * 10^−6^	3.3	[34]
K_A_	3.10 * 10^−4^	3.3	[34]
Na/K-ATPase	1.25 * 10^−4^	2.1	[34]
K_Leak_	6.37 * 10^−6^	Not applicable	Implemented in the current study

#### Extracellular field and temperature model

To resemble the cutaneous electrical stimulation, the extracellular field at the distal part of axon model was changed with the same shape as the current pulse applied through the electrode in the experimental study. Only the depolarizing part of the electrical pulse was used since there is no need to use a biphasic pulse to overcome the risk of damages to the skin in a computational model. For a section of 0.5 mm at the distal end of the axon model, the extracellular field was changed by the inbuilt function for an extracellular field in NEURON. The effect of the skin on the propagation of the electrical field was omitted. The extracellular field was increased until the axon model generated an action potential which propagates to the end of the model. The activation threshold was defined as the smallest extracellular field required to generate a propagating action potential.

For a section of 0.5 mm at the distal end of the axon model, the temperature was set to 20 °C. The temperature was then linearly increased over 4 mm of the model to reach 32 °C at the proximal section (see Fig. 1g). The time constants for the gating parameters are strongly influenced by temperature changes, but the steady-state parameters are less affected. Therefore, temperature dependencies of the steady-state parameters were omitted, except for the slow inactivation of the Na_TTXs_ current, since the slow inactivation gate is strongly influenced by temperature [23]. The slow inactivation gate of Na_TTXs_ was linearly shifted 20 mv towards hyperpolarization when the temperature was changed from 32 to 20 °C [23].

The maximal conductance is affected by changes in passive diffusion, a Q10 of 1.4 was also applied to all the ionic currents [38]. The temperature was linearly reduced from 32 to 20 °C during 2 s. The voltage-gated ion channels time constant and the Na/K-ATPase current temperature dependency were calculated by Eq. 2.

2 $$\begin{aligned} \hfill\uptau_{T2} = \tau _{T1} \cdot \frac{1}{{Q10^{{\frac{T2 - T1}{10}}} }} , \quad for\;all\; the \;ion \;channels \\ \hfill I_{T2} = I_{T1} \cdot Q10^{{\frac{T2 - T1}{10}}} , \quad for\; the\; Na/K \text{-}ATPase \\ \end{aligned}$$

Equation 2: *T*1 = temperature 1, *T*2 = temperature 2, I pump current, τ = time constant for the gating parameters.

#### Constraints of the computational model

The maximal conductances of the ionic currents were defined by the following constraints:

1. The axon model should display similar behavior for slowly increasing stimuli for both temperature conditions as the experimental data.
2. If an action potential is generated it should propagate to the end of the axon model.
3. The Na_TTXr_ current should generate the action potential [39].
4. The action potential should be higher than 0 mV.
5. If the Na/K-ATPase current is blocked (100%) the membrane potential should depolarize approximately 1.4–7 mV [25].
6. The K_Leak_ channel should be the major potassium background current [31].

#### Computational models of hyperexcitability

During several neuropathic conditions, the excitability of small fibers is increased [1, 2] but the ionic current generating the increased excitability is usually unknown. All three sodium channels included in the model, as well as the Na/K-ATPase, have been implicated with neuropathy in diabetic patients [40–44]. To generate four pathological models of hyperexcitability, the three sodium channels’ maximal conductances were increased, alternatively the Na/K-ATPase current was reduced in the computational model. The four computational models of hyperexcitability were:

1. The maximal conductance of Na/K-ATPase was reduced to 0.
2. The maximal conductance of Na_TTXr_ was increased 30%.
3. The maximal conductance of Na_TTXs_ was increased by a factor of 5.
4. The maximal conductance of Na_TTXs_ was increased by a factor of 2.

The abnormal ionic current alteration was changed after the balancing of the ionic current which could thereby influence the resting membrane potentials. The ionic currents which are active at resting potential may thereby influence the excitability by altering the resting membrane potential, for instance the Na_P_ and the Na/K-ATPase currents.

## Results

### Cooling reduced the perception threshold to small fiber stimulation in human

**Fig. 3:**
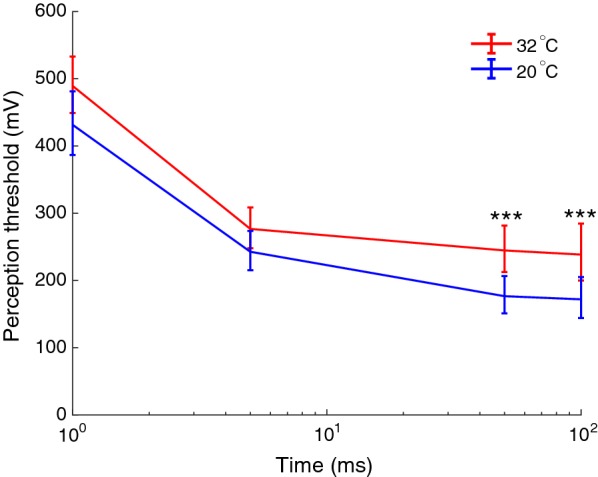
The effect of cooling the skin on the perception threshold. The experimental result of perception thresholds to electrical currents delivered to the skin with temperature regulated to 20 °C or 32 °C. Data is shown as mean and standard error. Statistically significant differences between the two temperature conditions are indicated by asterisks, ***p < 0.001, Sidak multiple comparisons

The PTT technique was used to assess the excitability related to slowly increasing electrical stimulation, simultaneously with controlling the skin temperature to either a cold 20 °C or a neutral 32 °C temperature by a thermode. When analyzing the perception thresholds, main effects of temperature (F(1,24) = 18.57, p < 0.001) and duration (F(3,72) = 32.73, p < 0.001) were found, as well as a significant interaction between the stimulation duration and temperatures (F(3,72) = 3.75, p < 0.001). The interaction was analyzed by a post hoc comparison, which revealed a significant decrease in perception threshold when stimulating with long pulse durations (50 and 100 ms) at 20 °C, compared to stimulating at 32 °C (p < 0.001) whereas for the shorter durations of the electrical stimuli, there was no significant reduction in perception threshold related to cooling (1 ms, p = 0.116; 5 ms p = 0.079, Sidak, Fig. 3).

### Cooling increased the excitability in the computational model

**Fig. 4:**
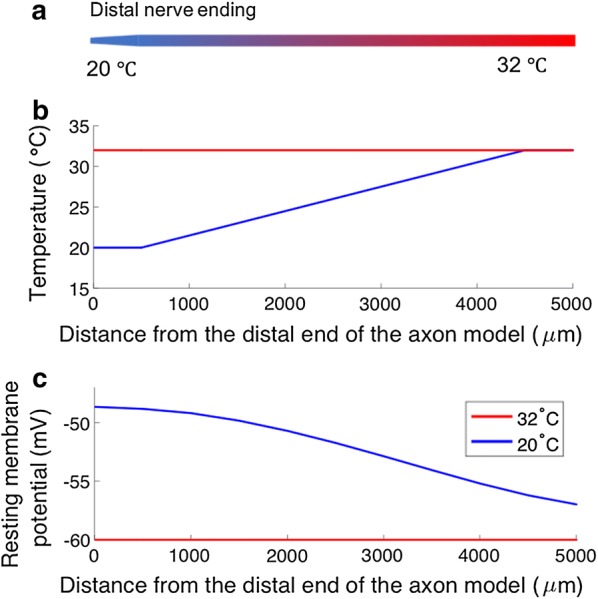
Depolarization of the resting membrane potential by reduced temperature. **a** The axon model design. **b** The temperature along the axon model for the two temperature conditions. The red and blue lines represent the temperature conditions 32 °C and 20 °C respectively. **c** The steady state solution of the membrane potential along the axon model. Note the large depolarization occurring at the distal end of the axon model (x axis = 0)

**Fig. 5:**
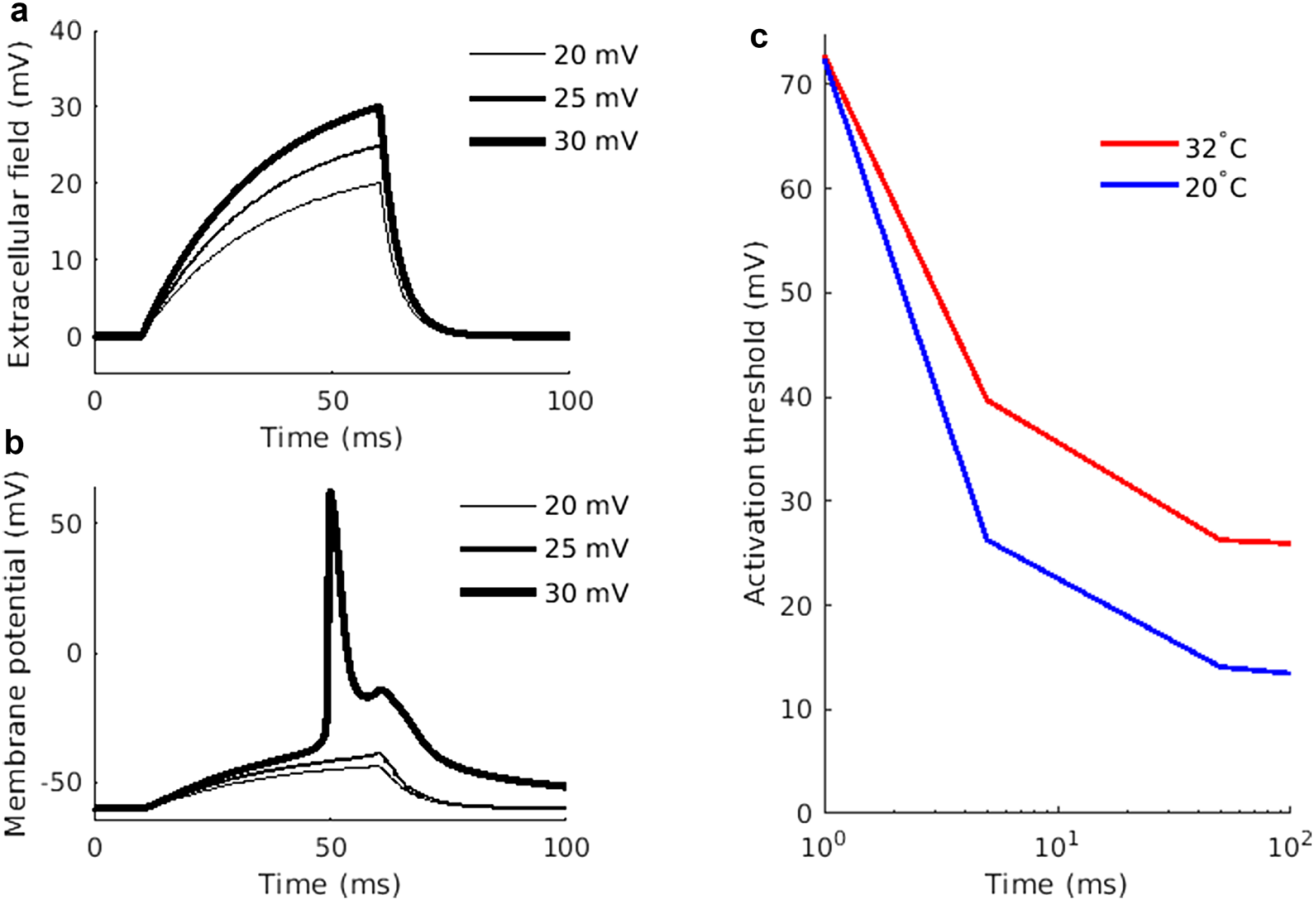
The experimental results could be reproduced in the computational model. **a** Extracellular field alteration potential at the distal end of the axon model. **b** The membrane potential at the distal end of the axon model induced when the alteration in extracellular field according to A was applied. **c** The activation threshold for different durations of the extracellular field alteration for the two temperature conditions. The activation threshold was defined as the extracellular field alteration required to generate and action potential propagating to the end of the axon model

To understand the excitability alteration occurring during cooling of the skin, a detailed multi-compartment model of an unmyelinated fiber was developed. The temperature was set to 20 °C at the distal (cold) section (0.5 mm) and linearly increased to 32°C along the axon model. The reduced temperature induced a 11.3 mV depolarization of the resting membrane potential (see Fig. 4b). To further study how the excitability is altered due to reduced temperature, the extracellular field along the distal section was altered (see Fig. 5a). The extracellular alteration led to a depolarization which—provided it was large enough—generated an action potential (see Fig. 5b). The activation threshold was defined as the lowest extracellular field alteration required to generate an action potential propagating to the end of the axon model. When the temperature was set to 32 °C, a 50 ms alteration of the extracellular field with an amplitude of 26 mV was required to generate a propagating action potential. When the temperature was reduced, a 14 mV change of the extracellular field was enough to generate an action potential (see Fig. 5c). The axon model displayed a reduced activation threshold for long durations of input and could thereby to a great extent reproduce the increased membrane excitability during cooling that was assessed by the PTT technique experiment (see Figs. 3 and 5c).

### How subtypes of ionic currents are affected by temperature reduction

**Fig. 6:**
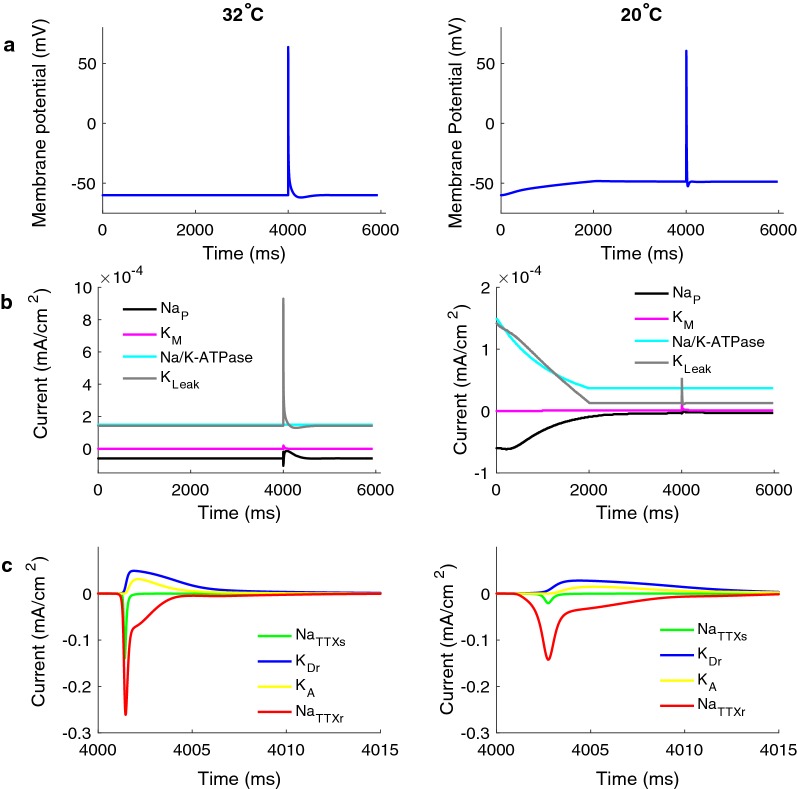
Ionic currents during an action potential generation. The extracellular field was altered for 1 ms with a bounded exponential shape. The figures to the left represent the simulation performed at 32 °C and to the right when the temperature is slowly decreased to 20 °C. **a** Membrane potential recorded at the distal end of the axon model. **b** The slow ionic currents. **c** The fast ionic currents

In Fig. 6, the action potential and the ionic current in the computational model are compared for the two temperature conditions 20 °C and 32 °C. Note the 11.3 mV depolarization of the membrane when the temperature is reduced (Fig. 6a). The depolarization is mainly due to the reduction of the Na/K-ATPase and the K_Leak_ current (see Fig. 6b). During cooling, all the ionic currents become slower, and the amplitudes are reduced (see Fig. 6). However, all amplitudes are not reduced equally for all currents, which in the computational model generated the increased excitability for the long durations of the stimulus.

**Fig. 7:**
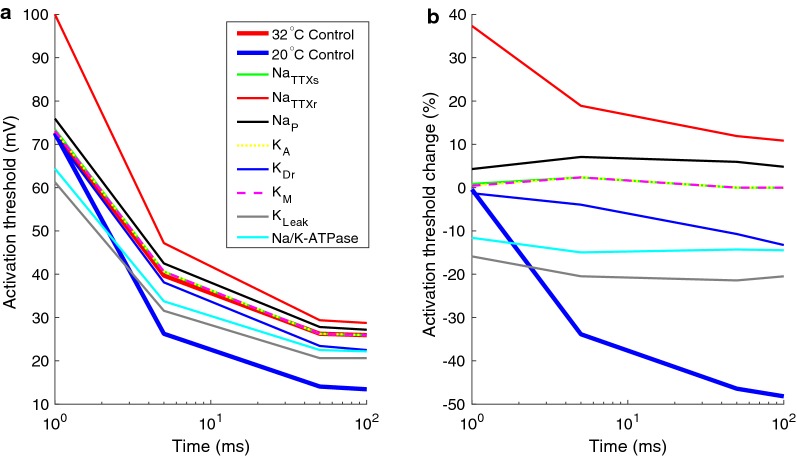
The influence of temperature on subtypes of ionic currents. **a** The activation threshold was estimated for each ionic current when temperature change was set to only influence that specific ionic current and no other currents. **b** The relative change of the activation threshold due to cooling when temperature change was set to only influence that specific ionic current and no other currents

To study the contribution to the altered excitability of different subtypes of ionic currents, only one ionic current at a time was allowed to be influenced by the temperature change (see Fig. 7). For instance, the light blue line in Fig. 7a represents a simulation when only the Na/K-ATPase dynamics were altered according to a temperature of 20 °C. All of the other ionic currents had their dynamics corresponding to a temperature of 32 °C. In Fig. 7b, the relative effect of the activation threshold is illustrated when an individual ionic current was influenced by the reduced temperature. The Na/K-ATPase and K_Leak_ currents generate a large reduction of the activation threshold for all input durations (approximately 14% and 20% respectively). The Na_TTXr_ generates a large increase in the activation threshold for the 1 ms and 5 ms durations (37% and 19% respectively). For the two longer durations (50 ms and 100 ms), the Na_TTXr_ current only increases the activation threshold by approximately 10%. By combining the effect of cooling on the Na_TTXr_ current with the Na/K-ATPase and the K_Leak_, the reduced perception threshold for long durations of the electrical input can be explained. Additionally, the K_Dr_ current may also contribute to the increase excitability to long durations of the electrical stimulation.

### Cooling may increase the understanding of abnormal ionic current during neuropathic conditions

**Fig. 8:**
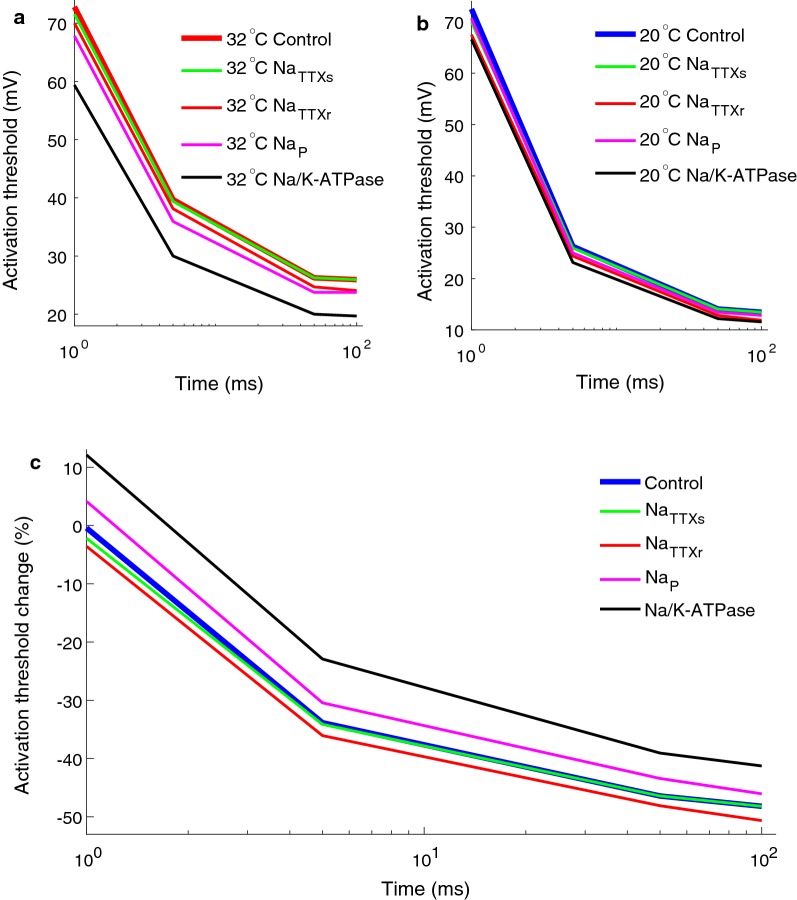
Cooling may increase understanding of abnormal ionic current during neuropathy. The Na/K-ATPase (0%), Na_TTXr_ (130%), Na_TTXs_ (500%) and Na_P_ (200%) maximal conductances were altered to generate the hyperexcitability models. **a** The activation threshold estimated in the hyperexcitability models and the control model (no alteration of the ionic conductances) for the temperature condition 32 °C. **b** The activation threshold estimated in the hyperexcitability models and the control model (no alteration of the ionic conductances) for the temperature condition 20 °C. **c** The relative difference between the activation threshold for the two temperature conditions (20 °C and 32 °C)

In pathological conditions, ionic currents may become abnormal and the excitability of nerve fibers related to reduced temperature may be altered. Increased knowledge of abnormal ionic currents in diabetic neuropathy patients may be obtained by comparing the perception thresholds, of different electrical stimulation, for different temperature conditions. To investigate this, four abnormal currents were implemented in the computational model: The Na_TTXr_, the Na_TTXs_, the Na_P_ and the Na/K-ATPase currents. All four ionic currents have been implicated in diabetic neuropathy and been proposed as candidates for generating the increased excitability detected in small fibers [40–44]. To generate four pathological models of hyperexcitability, the maximal conductances of the three sodium channels were increased, alternatively the Na/K-ATPase current was reduced. All ionic current alterations generated a reduced activation threshold for the temperature condition 32 °C (see Fig. 8a). The temperature was reduced in the computational models of hyperexcitability and the activation thresholds were re-estimated (Fig. 8b). For the temperature condition 20 °C, all models of hyperexcitability showed reduced activation thresholds compared to the control model. The relative change in activation threshold between the two temperature conditions for all models of hyperexcitability is illustrated in Fig. 8c. For instance, when the temperature was reduced in the model lacking the Na/K-ATPase current, the activation threshold was increased for the shortest pulse length (1 ms), whereas for the other three longer durations of the input (5 ms, 50 ms and 100 ms), only a small reduction of the activation threshold was recorded (Fig. 8c, black line). An increase of the Na_P_ current generated a similar behavior (Fig. 8, magenta line). Interestingly, the opposite behavior was displayed in the two models where the Na_TTXr_ and the Na_TTXs_ currents were increased i.e. cooling reduced the activation threshold more than during control conditions. A possible interpretation of these findings is that, if the diabetic patient’s perception threshold is increased for short durations of the electrical stimulation during cooling, this might indicate either that the Na/K-ATPase and/or the Na_P_ currents have been altered, but not the Na_TTXr_ or the Na_TTXs_ currents.

## Discussion

### The effect of cooling on small fiber membrane excitability

The study showed a reduced perception threshold when skin temperature was cooled from 32 to 20 °C for the 50 ms and 100 ms pulse durations. However, no significant effect was observed for the shorter pulse durations. The computational model indicated that depolarization occurred when the temperature was reduced. Both patch clamp experiment of small dorsal root ganglion somas, as well as threshold tracking of the compounded action potential of large nerve fibers in humans, support that a depolarization of the cell membrane occurs when the temperature is reduced [25–27]. This does not necessarily imply increased excitability because the slowing of sodium channels may counteract the increased resting membrane potential as observed for the short pulse durations in our study (1 and 5 ms). Since 50 ms and 100 ms stimuli are not in the range of normal receptor potential duration, the small fibers will rarely display increased excitability when the temperature is reduced under normal conditions. Although, long stimulation does not occur during normal fiber function, it can reveal knowledge regarding ionic currents which is amplified by slowly increasing currents, such as the Na_P_ and the Na/K-ATPase currents.

Moreover, the mechanisms for generating the depolarization of the cell membrane during a reduced temperature has not been identified. However, candidates have been proposed such the Na/K-ATPase, or the K_Leak_ channels [25–27]. In threshold tracking experiment of the compound action potential in large fibers, the excitability alteration due to temperature change has been studied [26, 27]. The excitability alterations induced by cooling in these studies were best explained by axonal depolarization due to the effect of temperature on Na/K-ATPase activity. This is consistent with our results from the computational model, which predict that a general depolarization related to cooling is generated by the Na/K-ATPase and/or a temperature dependent K_Leak_ channel, such as the TREK-1 [29]. The selective effect of cooling on long durations of the electrical stimuli may be due to increased time constants of the Na_TTXr_, which is selectively expressed in nociceptive fibers, where it generates the action potential [39]. In large fibers, non-nociceptors, the Na_TTXs_ current is generating the action potential in nodes of Ranvier. Interestingly the Na_TTXs_ current is more reduced during cooling than the Na_TTXr_ current (see Fig. 4). Therefore, cooling of the skin may increase excitability of nociceptors selectively and therefore promote preferential activation of nociceptors by cutaneous electrodes.

### The clinical relevance of the perception threshold tracking technique

Excitability of small fibers is usually studied through microneurography, which is technically challenging and time consuming due to the small fiber size. Our research group has developed the PTT technique, which is an inexpensive and non-invasive alternative method to indirectly assess the excitability of the cell membrane of both small and large fibers [10, 17]. In the current study, it was possible to detect the excitability change due to reduced temperature with the PTT technique and thereby increase the support for the usage of the PTT technique to assess the membrane excitability in nerve fibers. Moreover, the result from the computational study supports that cooling simultaneous with electrical stimulation may increase the knowledge of abnormal ionic membrane currents occurring in neuropathic pain patients. For instance, animal models of diabetic neuropathy have shown that the Na/Ka-ATPase is impaired due to the altered metabolism occurring in this patient group [40, 43]. It would be interesting to evaluate whether the reduced perception threshold for long pulse durations due to cooling would be less reduced in this patient group compared to healthy participants as studied in the current study. If that would be the case, it would support the hypothesis of an impaired Na/K-ATPase current.

### Electrical stimulation parameters and planer array electrode used in the experiment

The perception threshold to a slowly rising electrical pulse was measured for durations from 1 to 100 ms delivered through a planar array electrode. The pulse had an increasing form of bounded exponential current, which for long durations has been shown to elevate the perception threshold of large fibers but not for small fibers [17]. This most likely reflects accommodation of large fibers, which has been reported for identical electrical pulse shape [45] and linearly increasing pulses [46]. It should be noted that for standard rectangular pulses, large fibers are recruited prior to small fibers [47] and therefore, the pulse shape applied in current study is considered more preferential towards the small fibers. The preferential activation of small fibers with the planar array electrode has not been evaluated. However, similar small area cathodes have been used to activate small fibers preferentially [5, 7, 9, 10, 17]. Additional support for the Planar array electrode preferential activation of small fibers is the lack of accommodation to slowly rising electrical pulses. The basis for the use of the Planar array electrode in the current study was its flat dimensions, which makes it possible to use a thermode to regulate the temperature, which is an essential part of this study. This feature separates it from currently available pin electrodes, as the dimensions of these electrodes do not allow for the thermode to be placed on top of the electrode for cooling.

### The influence of cool-sensitive transduction channels on the results

In the current study, the influence of reduced temperature on nerve fiber excitability is investigated, with as little influence as possible from cool-sensitive transduction channels within sensory terminals. Instead, our focus is on the effect of cooling on other ionic currents in the peripheral part of small fibers by using electrical stimulation as test stimulus. The recorded alterations of perception thresholds to the electrical pulses in the current study are dependent on the duration of the electrical input. It is therefore reasonable to assume that the electrical stimulation, rather than the reduced temperature, mainly activated the fibers.

Cutaneous electrical stimulation alters the membrane potential without activation of sensory transduction within sensory terminals. Nociceptive Aδ fibers are mainly activated during transcutaneous electrical stimulation with small cathode electrodes [5, 9, 17]. Cold sensation has to the authors’ knowledge not been reported in studies using this type of cutaneous electrical stimulation [3, 8, 17]. Small cathode electrodes generate a high current density in epidermis where the small fibers terminate [11–16]. Therefore, the nerve fibers’ proximity to the cathodes and the shape of the electrical pulse will influence whether the fiber will become activated by the stimulus or not. The small influence on the perception threshold from cold sensing fibers can be explained by the fact that only a small proportion of the fibers express the cool-sensitive transduction channels.

By reducing the temperature to 20 °C, cold sensitive non-noxious fibers will indeed become activated and cool-sensitive transduction channels could produce a significant depolarization, which could influence the perception threshold. The question is whether the population of cold non-noxious Aδ fibers is sufficient to influence the perception threshold. A recent study by Luiz et al. [48], showed that non-nociceptive cold-activated small fibers do not express Na_TTXr_ (Nav1.8). Since 93% of the Aδ fibers due express Na_TTXr_ (Nav1.8) in the DRG somas of rats [49], this leads to the conclusion that less then 7% of the Aδ fibers are non-nociceptive cold sensing fibers. Therefore, only a small part of the depolarization predicted by the computational model could originate from cold sensing nerves.

### Limitations

The perception threshold was used as an indirect measurement of the excitability of the cell membrane. To avoid sensitizing the central nervous system low frequency and intensity was used in the current study since high frequency and intensity has been used to induce long term potentiation-like sensitization of the central nervous system [7]. Other central nervous system effects such as expectation and attention are probably the same for the different durations of the electrical stimuli. The repeated low intensity pulses might have caused habituation as observed in a recent study [50]. Therefore, to overcome this limitation, the order of pulse durations was randomized, as well as the two temperature conditions. Habituation also occurs to the cold stimulus, but is likely limited due to relatively high temperature applied in the current study [51].

One of the limitations of the computational model is that the axon model has a reduced morphology, which may affect the validity of the model results. Furthermore, the computational model did not include the influence of the electrical properties of the skin.

## Conclusion

The study showed that cooling of the skin decreased the perception threshold particularly for the long duration of slowly increasing pulses. The results of the computational model predicted that the perception threshold reduction originated from a reduction of the K_Leak_, the Na/K-ATPase and the Na_TTXr_ current. Cooling may alter the ionic current during electrical stimulation and thereby provide additional information regarding membrane excitability of small fibers. The PTT technique could detect the excitability alteration occurring during reduced temperature and may have the ability to become a diagnostic tool for neuropathy.

## Data Availability

The datasets used and analyzed during the current study are available from the corresponding author on reasonable request.

## Abbreviations

PTT: perception threshold tracking
Na_TTXs_: TTX sensitive sodium channel (mainly Na_v_1.7)
Na_TTXr_: TTX resistant sodium channel (Na_v_1.8)
Na_P_: persistent sodium channel (Na_v_1.9)
K_A_: fast transient potassium channel
K_Dr_: delayed rectifier potassium channel
K_M_: slow potassium channel
K_Leak_: leak potassium channel
